# Supplementary material for: Unintentional injuries and potential determinants of falls in young children: Results from the Piccolipiù Italian birth cohort
Source: PLoS One. 2022 Oct 3;17(10):e0275521. doi: 10.1371/journal.pone.0275521 (PMC9529104; doi:10.1371/journal.pone.0275521)
Supplement: S1 Table — (PDF) [file pone.0275521.s001.pdf]

S1 Table

	log-rank test statistic	*p-value*
Enrollment area	33,25	0,00
Child gender	1,39	0,24
Maternal age at delivery	11,05	0,00
Maternal education	0,44	0,80
Maternal employment	0,01	0,93
Paternal employment	1,46	0,23
EHII	2,12	0,35
Number of siblings	0,53	0,47
Day care attendance	0,27	0,60
Maternal distress	24,75	0,00
Maternal smoking during pregnancy	3,61	0,06
Maternal alcohol intake during pregnancy	11,45	0,00
Time needed to fall asleep	2,79	0,09
Sleeping problems (reported by parents)	12,32	0,00
Where the child sleeps	6,71	0,03
The child use a comfort object for sleeping	0,02	0,89
