# Supplementary material for: Unintentional injuries and potential determinants of falls in young children: Results from the Piccolipiù Italian birth cohort
Source: PLoS One. 2022 Oct 3;17(10):e0275521. doi: 10.1371/journal.pone.0275521 (PMC9529104; doi:10.1371/journal.pone.0275521)
Supplement: Attachment — Submitted filename: Response to Reviewers.docx [file pone.0275521.s002.docx]

**Editor Comments:**

Dear authors, please describe more thoroughly how the multivariable model was built after selection of variables in the univariate analysis. This procedure is not fully described and it is difficult to reproduce by others as it stands now. It is unclear what the criteria for retaining and removing variables from the multivariable model were; similarly, no test for predefined interaction terms (if any) are mentioned. Please also reconsider the use of a p-value 0f 0.05 for variable selection in the univariate analysis; by using this threshold of significance, you may lose important information at the stage of variable selection. Please refer to the Hosmer, Applied Logistic Regression, as example foe how predictor model building should be performed. It is also unclear how multicollineraity was evaluated; it seems that was examined between covariates, before model building - this step should be clearly described.

RE: Dear editor, we want to thank the editor and the reviewers for the useful suggestions that we believe significantly increased the quality of the Manuscript. Regarding the issue of selection of the predictor variables considered for the multivariable model we preferred to test each potential predictor at a time, before to include it into the multivariable model. In the first step we aimed to select the predictors with a stronger association with our outcome using a log-rank test with a p-value <0.05 as inclusion criteria, since we are in a Cox model setting. About the choice of the threshold of significance, we preferred to follow a conservative approach above all, because of the big number of predictors that we had (as reported in ‘Potential risk factors’ paragraph) in order to build a Cox regression model that included the minimum set of predictors thus avoiding over adjustment and multicollinearity. Moreover, since there is not a gold standard for the cut-off to identify predictor as “statistically significant” associated to the outcome, we think this cut-off (p<0.05) was able to discriminate among many competing hypotheses, selecting those more compatible with the data. (Greenland et al. 2016, doi: [10.1007/s10654-016-0149-3](https://doi.org/10.1007%2Fs10654-016-0149-3); Miller et al. 2019, https://doi.org/10.1371/journal.pone.0208631).

In the second step we tested for independence among predictors, resulted significant in the first step, using a Chi-Square test since all predictors were qualitative. In the third step we run the explicative multivariable Cox proportional hazards model including all predictors significantly associated to the outcome and independent among each other. In the final model we did not use a stepwise method for retaining or removing variables and we did not test interaction terms between covariates.

To check the presence of multicollinearity in the final model we used the variance inflation factor (VIF), considering the presence of collinearity when VIF was higher than 10.

We modified accordingly the Method section in the lines 144-157.

Missing values on covariate: please indicate the overall extent of missing values on covariates in the study, and indicate how they were handled in the analysis. It seems that a listwise deletion approach was used. If this is the case, please acknowledge the limitation of having such an approach on your results as compared to more robust methods such as multiple imputation (depending on the assumption as to why data are missing).

RE: As for missing values of covariates we reported the “non- responders” in the Table 3. Since the percentages of the missing values in the covariates were between 0.1% and 6.5% (with the only exception of a complex index of maternal distress with a missing value of 10%), we used a complete-case approach.

We added accordingly a sentence on this aspect at page 7 line 155-157.

**Reviewers' comments:**

Reviewer #1: It is valuable to build high-quality cohorts to support examining important research questions for injury prevention. Nevertheless, however, this research did not justify several critial issue this their injury cohort.

1. It seems that the authors did not adequately justify the rationale of implementing the Piccolipiù Italian birth cohort. Specifically, it is unclear the innovations of this cohort compared to the published cohorts related to injury prevention. Consequently, the

research questions to be addressed by the Piccolipiù Italian birth cohort are unclear.

RE: We thank the reviewer for this observation. We better clarified that epidemiological injury research has mostly used routine data sources and registries. Moreover, some work has used birth or child cohorts which, however, were mainly performed in Northern European countries (lines 60-68).

2. The raised surface fall (FERF) was not clearly defined in the manuscript.

RE: FERF is an acronym for “**F**irst **E**vent of **R**aised Surface **F**all” during the first two years of life; we have now written the acronym in capital letters (line 71).

3. The selection of potential risk factors and their operational definitions was not clearly described. This is particularly important for addressing high-quality research questions.

RE: Regarding the selection of the predictor variables considered for the multivariable model we preferred to test each potential predictor at a time, before to include it into the multivariable model. In the first step we aimed to select the predictors with a stronger association with our outcome using a log-rank test with a p-value <0.05 as inclusion criteria, since we are in a Cox model setting. In the second step we tested for independence among predictors, resulted significant in the first step, using a Chi-Square test since all predictors were qualitative. In the third step we run the explicative multivariable Cox proportional hazards model including all predictors significantly associated to the outcome and independent among each other.

According to the request of the reviewer, the selection of predictor variables included in this study have been described in more details (lines 144-157).

4. In addition, it is hard to understand the "raised surface falls reported" among children within 6 months. Because we know such young children cannot independently walk. Please explain the reason for it.

RE: Falls are the most common mechanism of unintentional injury but of course injury’s mechanisms change with age. In this study falls from raised surface that affected children in the first and second year of life were described. In Table 2 (line 170) it is reported the place (eg. bed, chair, table...) from where the child fell in the first 12 months of life (period in which we can assume that the child is not yet able to independently walk). Results show that falls from a raised surface occurred more frequently from the bed (57%), followed by table/chair/high chair (11.6%), changing table (6%) and etc.

Reviewer #2: This paper describes unintentional injuries and risk factors of falls in young children from a Piccolipiù Italian birth cohort. This paper addresses an important topic in a niche population. However, it would benefit from restructuring, strengthening the introduction, distinguishing the results and the discussion, and carefully reading through for any grammatical and clarity concerns.

RE: We thank the reviewer for pointing this out. We have accepted your suggestions and the final manuscript was revised by a native English speaker.

Abstract
• Include any hypothesized findings

RE: We are not sure to have understand this comment; anyway, we added a paragraph in the Abstract to clarify why unintentional injuries are important to be investigated in birth cohorts such as Piccolipiù (lines 22-25).

Introduction
• The introduction starts off strong but weakens near the end. The structure somewhat falls apart and some of the implications of the study are lost.

RE: We thank the reviewer for this comment. We modified the text according to the suggestions (lines 60-68).

• Lines 58-60 on page 3 are a little confusing and could be reworded.

RE: We thank the reviewer for these comments. We modified the text according to the suggestions (lines 60-68).

• Why are we specifically describing injuries in this cohort and in Italy? I think the introduction would benefit for more context on why this study is relevant and how it can be helpful to this population.

RE: We thank the reviewer for this comment. The Introduction has been enriched with more information from the literature available in this field (lines 60-68).

Methods

• I’m a little confused about the meaning of “Piccolipiù.” It’s described as a “birth cohort” but what does that mean in this context? At first I though it was a city in Italy but that does not seem accurate either. Further clarity on this is essential to really understand the population of this paper.

RE: We thank you for this comment. We clarified in the text that Piccolipiù is the name of our birth cohort (line 75).

• Do you have any examples that can be provided regarding the details about the raised surface falls?

RE: In this study, falls from raised surface that affected children in the first and second year of life were described. In Table 2 were reported some details about the age of child, the place where the fall happened (bedroom, living room, nursery etc),the place from where the child fell (bed, table/chair/high chair, changing table, arms, etc), the person who was with the child (mother, father, grandparents, siblings, baby sitter, etc), the type of injury reported (bruise, bleeding, blow, etc) and the treatment required (medical examination, emergency hospital department, hospitalization, etc).

• Do you have any psychometrics to report on the EHII?

RE: The Equivalised Household Income Indicator (EHII), was used as indicator of the total disposable monthly household income at birth standardized by household size and composition. It was derived using Piccolipiù cohort data (maternal age, cohabitation status, country of birth, educational level, occupational status and occupational code; paternal/partner age, country of birth, educational level, occupational status and occupational code; and household size and tenure status) and external data from the Italian 2011 “European Union Statistics on Income and Living Conditions” (EU- SILC) survey. This is definitely an interesting aspect but this is not the focus of this article.

• On page 5, the line about children’s sleeping behavior (lines 121-123) is confusing. And why did it get dichotomized?

RE: Children’s sleeping behavior was assessed by considering several items of the questionnaire. One of them is the parental perception of child’s sleeping behavior which was a categorical variable in the questionnaire with 3 levels. We decided to recode this variable in a dichotomous way (no/yes), collapsing in “yes” the categories 2=”somewhat a problem” and 3=”quite a problem”, since they had low frequencies.

• Lines 128-134 feel out of place in the methods section. I recommend moving them to the Statistical Analysis section.

RE: We thank the reviewer for pointing this out. We deleted this paragraph and reworded it with a detailed explanation of the entire process of variables selection in the Statistical Analysis section (lines 144-149).

Statistical Analysis
• Lines 137-142 should be placed in the Methods section.

RE: These lines are already in the Method Section (sub-section “Statistical Analysis”). We think they are important to understand the type of analysis performed and how the model was built. We prefer to maintain this part in the same place.

• I don’t understand what is meant by the following: “Children without documented falls at the end of the study were censored at the date of the last available questionnaire.” What does “censored” mean in this context?

RE: Censoring is referred to subjects who have not achieved any fall from raised surface. For example, if a child didn’t have any event of fall and had a questionnaire filled in at 24 months (the end of study period or follow-up), the time to event considered was 24.

Results
• Page 7, lines 152-153 need to be revised as they are currently confusing: “Unintentional injuries occurred to children of the Piccolipiù cohort in the first and in the second year of life are presented in Table 1.”

RE: We slightly modified this sentence to make it clearer and more understandable (lines 160-161).

• For Table 2, why were some categories multiple choice and not others?

RE: The questionnaires at 12 and 24 months of children’s age included some specific questions regarding falls from raised surface which occurred in the first and in the second year. For all questions only one answer was admitted, except for the question related to the type of injury (since many children had more than one type of injury), as reported in Table 2.

• This section overall needs improved set up and structure. It is difficult to follow the separate ideas because of the current presentation.

RE: We agree with the reviewer and we added some sentences in order to improve this section (lines 160-161, 164, 166, 181, 193-195).

Discussion
• Why might younger mothers be at higher risk? There is no interpretation of this finding.

RE: We hypothesize that younger mothers could have a lower risk perception and therefore they could pay lesser attention to prevent children’s unintentional injuries. We explained better this concept in lines 212-214.

• Any thoughts on the contrast in findings listed on page 12 lines 118-221? Also, is that supposed to be a separate paragraph? It seems randomly placed.

RE: We thank the reviewer for pointing this out. We tried to highlight that these different findings, obtained in a study conducted in UK, could be due to a different study population, since Mulvaney and Kendrick [ref 32], differently from our study which have been performed in general population, enrolled only mothers living in socio-economically disadvantage areas (line 226).

• I am not sure what is meant for lines 231-232 (page 13), can you elaborate further?

RE: We found that children living in northern cities of Italy (Turin and Trieste) are at lower risk of FERF compared to those living in central cities (Rome, Florence and Viareggio). This result is somewhat difficult to explain with the data at hand but we could hypothesize that this could be attributable to different safety behavior of parents who live in different cultural contests (lines 239-242)

• The limitations section can benefit from adding what can be done differently next time and/or why the limitation existed to begin with.

RE: We think the self-collected data used in the study could be one of the major limitations due to high risk of information bias (eg. underreporting of injuries) according to child’s health, family characteristics and environmental variables (eg. educational level, parity, household density). Moreover, a questionnaire administrated by a properly trained interviewer could definitely reduce the amount of missing data. We added a paragraph in lines 258-260.

• Consider adding some of the reported findings currently in the discussion to the results section. Right now the discussion feels too much like the results.

RE: We agree with the reviewer and have shortened the discussion section.

Conclusion
• Could benefit from a comment on any intervention being done to address these concerns.

RE: We agree with the reviewer and we better clarified the kind of interventions could be done to reduce the unintentional injuries in early life. Since the role of parents in maintaining toddler’s safety is crucial, we think these interventions should especially promote environmental safety and supervision of the child, therefore supporting parents from the birth or during birth classes (lines 274-276).
