# Supplementary material for: Unintentional injuries and potential determinants of falls in young children: Results from the Piccolipiù Italian birth cohort
Source: PLoS One. 2022 Oct 3;17(10):e0275521. doi: 10.1371/journal.pone.0275521 (PMC9529104; doi:10.1371/journal.pone.0275521)
Supplement: Attachment — Submitted filename: PLOS-ONE R.1.docx [file pone.0275521.s003.docx]

This paper remains to be critical and meaningful to the unintentional injury literature, but there are still some issues with the quality of writing and structure of the paper when considering clarity, reproducibility, and transparency about the data and statistical analyses.

**Introduction**

- The introduction could still benefit from added context about the significance of the research questions and research population. Please reevaluate the current literature included in the introduction and consider a section that focuses on the regions of interest.
- There remains some issues with clarity. Although I appreciate the effort put into improving lines 58-60 on page 3, it continues to be difficult to follow. It is clear that this is an important point so please take the time to revise and improve clarity.

**Methods**

- I appreciate the authors’ considerations of revisions for this section and especially am grateful for the additional information provided within the statical analysis section. However, there are some big issues that continue to remain.
  - The sleeping behavior paragraph continues to be difficult to follow. The response to the reviewers was helpful and should potentially be integrated into the text.
  - Although I understand the EHII is not the focus of the paper, given that it is not a standardized measure, it is relevant and critical to include psychometric properties to help the readers understand the validity and reliability of the measure.
  - I appreciate the clarification of “censored” but do not agree it is the best way to describe what happens to those participants. Please review what you wrote in the response to the reviewer and consider if it can be better integrated in the text.

**Results**

- I appreciate the attention provided by the reviewers toward revisions in this section but believe it would benefit from additional revisions. Regarding the differences in questionnaires and items (e.g, some were multiple choice and some were not), the authors might consider explaining this more clearly earlier on in the methods so that it can be more easily understood in the results.
- Overall, the structure of this section is much more easy to follow.

**Discussion**

- Although I appreciate the interpretation about young mothers, I want to know why this may be the case. Do you have any citations to demonstrate young mothers might have lower risk perceptions? Or any theory as to why? I know this is not the main purpose of the paper but when considering interpretations provided in the discussion, think about how to best explaining the findings in your results because that information is most helpful to the readers. You do a great job of this with the maternal psychological distress comments.
- Lines 243-247 are very difficult to follow, please revise for clarity.
- The authors did a good job of addressing revisions to the limitations and conclusion sections.
