# Supplementary material for: Unintentional injuries and potential determinants of falls in young children: Results from the Piccolipiù Italian birth cohort
Source: PLoS One. 2022 Oct 3;17(10):e0275521. doi: 10.1371/journal.pone.0275521 (PMC9529104; doi:10.1371/journal.pone.0275521)
Supplement: Attachment — Submitted filename: Response to Reviewers.docx [file pone.0275521.s004.docx]

**Comments to the Author**

5. Is the manuscript presented in an intelligible fashion and written in standard English?

PLOS ONE does not copyedit accepted manuscripts, so the language in submitted articles must be clear, correct, and unambiguous. Any typographical or grammatical errors should be corrected at revision, so please note any specific errors here.

Reviewer #1: No

Reviewer #2: No

RE: As suggested the entire text of the article was revised by a native English speaker.

6. Review Comments to the Author

Please use the space provided to explain your answers to the questions above. You may also include additional comments for the author, including concerns about dual publication, research ethics, or publication ethics. (Please upload your review as an attachment if it exceeds 20,000 characters)

Reviewer #1: Thanks for the replies to my comments. However, I don't think the replies and revisions towards comments 1 and 3 that I raised are adequately addressed. In this case, the innovations of this paper look to be weak.

RE: We thank the reviewer for this observation. We tried to improve the Introduction section in order to clarify that our cohort could contribute to increase the knowledge in this field of research (lines 63-69). We have therefore also reviewed the literature, adding several new references.

Regarding the third comment previously mentioned, to define the set of potential predictors to be included in the multivariable model, we considered some a priori risk factors based on literature (such as: sex, maternal age, maternal distress) and some risk factors based on biological plausibility, for example child sleeping behavior. All risk factors included in this study were assessed trough questionnaires.

Reviewer #2: This paper remains to be critical and meaningful to the unintentional injury literature, but there are still some issues with the quality of writing and structure of the paper when considering clarity, reproducibility, and transparency about the data and statistical analyses.

Introduction
• The introduction could still benefit from added context about the significance of the research questions and research population. Please reevaluate the current literature included in the introduction and consider a section that focuses on the regions of interest.

RE: We thank the reviewer for this observation. We tried to improve the Introduction section in order to clarify why our cohort could contribute to increase the knowledge in this field of research (lines 63-69). We have therefore also reviewed the literature, as suggested, adding several new references.

• There remains some issues with clarity. Although I appreciate the effort put into improving lines 58-60 on page 3, it continues to be difficult to follow. It is clear that this is an important point so please take the time to revise and improve clarity.

RE: We thank the reviewer for this observation. We better clarify this paragraph in lines 63-69.

Methods
• I appreciate the authors’ considerations of revisions for this section and especially am grateful for the additional information provided within the statical analysis section. However, there are some big issues that continue to remain.

o The sleeping behavior paragraph continues to be difficult to follow. The response to the reviewers was helpful and should potentially be integrated into the text.

We thank the reviewer for this comment and we added an explanation in the text as suggested (lines 141-143).

o Although I understand the EHII is not the focus of the paper, given that it is not a standardized measure, it is relevant and critical to include psychometric properties to help the readers understand the validity and reliability of the measure.

RE: We thank the reviewer for pointing this out. We have not understood the meaning of psychometric properties. However, the EHII is a measure of the total disposable household monthly income equivalised for household size and composition (currency: log-euro).

This income measure is obtained with a prediction model applied to the EUSILC data (Italian EUSILC survey for Piccolipiù) including as predictors the following variables (maternal age, maternal cohabitation status, maternal country of birth, maternal educational level, maternal occupational status and maternal occupational code; paternal age, paternal country of birth, paternal educational level, paternal occupational status and occupational code, household size and tenure status). This income measure has been derived for approximately 20 European birth cohort studies within the framework of the H2020 LifeCycle project. As described in the paper by Pizzi et al, the prediction models for each study have been already validated (temporal validation) and the derived measures compared with other SEP-related questionnaire-based information, including self-reported income, showed a strong correlation.

o I appreciate the clarification of “censored” but do not agree it is the best way to describe what happens to those participants. Please review what you wrote in the response to the reviewer and consider if it can be better integrated in the text.

We thank the reviewer for this comment and we added an explanation in the text as suggested (lines 151-152).

Results

• I appreciate the attention provided by the reviewers toward revisions in this section but believe it would benefit from additional revisions. Regarding the differences in questionnaires and items (e.g, some were multiple choice and some were not), the authors might consider explaining this more clearly earlier on in the methods so that it can be more easily understood in the results.

RE:
We thank the reviewer for this comment and we added an explanation in the text as suggested (lines 100-101)

Discussion
• Although I appreciate the interpretation about young mothers, I want to know why this may be the case. Do you have any citations to demonstrate young mothers might have lower risk perceptions? Or any theory as to why? I know this is not the main purpose of the paper but when considering interpretations provided in the discussion, think about how to best explaining the findings in your results because that information is most helpful to the readers. You do a great job of this with the maternal psychological distress comments.

RE: We thank the reviewer for this observation. Some demographic risk factors for childhood injury were already identified in different studies and of course the maternal age is one of them. Our result about the association between maternal age and FERF is very consistent with results from other studies. In the systematic review of Mitton et al., which identified cohorts of school-aged children and adolescents, is hypothesized that younger mothers, compared with older, may be less aware of the risks that children encounter as they develop and grow (Mitton et al. 2008). We added a sentence about this in order to better explain this concept (lines 221-225)

• Lines 243-247 are very difficult to follow, please revise for clarity.

RE: We thank the reviewer for this comment and we better explain the concept (lines 247-249).
